# Estimation of Cachexia among Cancer Patients Based on Four Definitions

**DOI:** 10.1155/2009/693458

**Published:** 2009-07-01

**Authors:** Kathleen M. Fox, John M. Brooks, Shravanthi R. Gandra, Richard Markus, Chiun-Fang Chiou

**Affiliations:** ^1^Strategic Healthcare Solutions, LLC, P.O. Box 543, Monkton, MD 21111, USA; ^2^College of Pharmacy, University of Iowa, Iowa City, IA 52242, USA; ^3^Amgen Inc., Thousand Oaks, CA 91320, USA

## Abstract

*Objectives*. Estimate and compare the proportion of cancer patients with cachexia using different definitions from available clinical data. *Methods*. Electronic medical records were examined to estimate the proportion of cancer patients with cachexia using 4 definitions: (1) ICD-9 diagnostic code of 799.4 (cachexia), (2) ICD-9 diagnosis of cachexia, anorexia, abnormal weight loss, or feeding difficulties, (3) prescription for megestrol acetate, oxandrolone, somatropin, or dronabinol, and (4) ≥5% weight loss. Patients with cancer of the stomach, pancreas, lung, colon/rectum, head/neck, esophagus, prostate, breast, or liver diagnosed between 1999 and 2004 were followed for cachexia. *Results*. Of 8541 cancer patients (60% men and 55% Caucasian), cachexia was observed in 2.4% of patients using the cachexia diagnostic code, 5.5% expanded diagnoses, 6.4% prescription medication definition, and 14.7% with ≥5% weight loss. *Conclusions*. The proportion of patients with cachexia varied considerably depending upon the definition employed, indicating that a standard operational definition is needed.

## 1. Introduction

Cachexia, often referred to as the wasting disease, is a complex metabolic syndrome associated with underlying illness and characterized by loss of muscle with or without loss of fat mass [1, 2]. However, the prominent clinical feature of cachexia is weight loss with anorexia, inflammation, insulin resistance, and increased muscle protein breakdown as additional frequent features [3, 4]. Patients with gastric, pancreatic, colorectal, or lung cancer often experience significant weight loss, including loss of skeletal muscle mass and develop cachexia with or without anorexia [5–7]. It has been shown that at the time of diagnosis, 80% of patients with upper gastrointestinal cancer and 60% of patients with lung cancer have already experienced a significant weight loss [8]. 

Cachexia is estimated to be the immediate cause of death in 20% to 40% of cancer patients [1, 8]. Several investigations have provided evidence that cancer patients with significant weight loss also report impaired quality of life [9–11], decreased response to chemotherapy [7, 12], and more frequent and severe toxicity [12]. The specific etiology of cancer cachexia is not entirely understood since cachexia can manifest in individuals with metastatic cancer as well as in individuals with localized disease. Cachexia also does not appear to be the result of tumor size, type, or extent [5]. 

However, due to its complex pathology and clinical presentation, cachexia may be underdiagnosed. There is no single, generally agreed-upon definition of cachexia [13, 14]. Since no standard definition of cachexia is available, cachexia is infrequently identified, or diagnosed and rarely treated [13, 15]. There are no approved medications to treat cachexia and its muscle wasting/loss apart from growth hormone and some appetite stimulants [14]. Hence, physicians treat the symptoms of anorexia, weight loss, and insulin resistance. Additionally, physicians do not routinely assess or measure muscle loss in their patients even though loss of muscle mass is a hallmark sign of cachexia. Physicians may note weight loss, weakness or fatigue in the patient's medical record and the patient may be given a diagnosis of anemia or anorexia instead of cachexia. Thus, accurate estimates of the prevalence of cachexia among cancer patients are limited. It is essential to have a validated and universally accepted definition of cachexia so clinicians can recognize the problem and institute treatment [13, 14]. This retrospective database study of electronic medical records was designed to examine different definitions of cachexia using available clinical data sources and determine the variation in the proportion of patients with cachexia using four definitions of cachexia. The study objectives were to quantify the number of patients identified with cachexia among cancer patients and to characterize these patients with cachexia compared with cancer patients without cachexia. The study was not designed to identify the best definition or criteria but rather to understand the potential variability in cachexia and patient characteristics.

## 2. Methods

A retrospective database study of electronic medical records was conducted to ascertain the variation in the proportion of cancer patients with cachexia using four definitions of cachexia: (1) ICD-9 code for cachexia (ICD-9-CM 799.4), (2) ICD-9 code for cachexia, ICD-9-CM 783.0 for anorexia, ICD-9-CM 783.2x for abnormal weight loss, or ICD-9-CM 783.3 for feeding difficulties, (3) at least one prescription for megestrol acetate, oxandrolone, somatropin, or dronabinol, and (4) ≥5% weight loss postcancer diagnosis. The ICD-9 diagnostic code of 799.4 states cachexia or wasting disease with no definition of cachexia provided. The first occurrence of any one of the definition criteria was defined as the presence of cachexia. Since there is no consensus in the field on the definition of cachexia, 4 different definitions were used to understand the range in frequency of cachexia. These definitions were chosen since the data would be readily available in clinical practice and administrative databases.

All eligible cancer patients from the Henry Ford tumor registry diagnosed between 1999 and 2004 were assessed for the presence of cachexia, using each of the four study definitions. Data from the Henry Ford Health System tumor registry and electronic medical records was utilized to identify cancer patients and assess the characteristics of patients with cachexia. Henry Ford Health System (HFHS) is one of the leading US vertically integrated health care systems providing healthcare services to the Michigan and Ohio region. The tumor registry includes information on nearly 2500 newly diagnosed cancers annually, including primary site, histological type, disease stage, and treatment course. Electronic medical records were merged for eligible cancer patients to obtain data on demographics, weight, and comorbid conditions.

Eligible cancer patients included the following cancer types: gastric, pancreatic, lung (nonsmall cell and small cell), colorectal, head or neck, esophageal, prostate, breast, hepatic or liver. These cancer types were included because estimates from the US Cancer Statistics Working Group indicated that 85% of gastric cancer, 83% of pancreatic cancer, and 61% of nonsmall cell lung cancer patients exhibit significant weight loss (≥5%), loss of muscle mass, and develop cachexia, with or without the presence of anorexia [7]. Patients with no tumor stage and those with a prior history of cancer (as noted in patient's admitting history and physical) were excluded from the study. Patients were followed from date of cancer diagnosis to death, date of drop-out or end of study (December 2006) to determine the presence of cachexia. For the ICD-9 diagnostic codes and prescription medication definitions, a patient was identified as having cachexia if the diagnosis or medication was present at the time of or after the cancer diagnosis to ensure that all prevalent cases of cachexia were identified. This could not be done for the weight loss definition because weight prior to cancer diagnosis was not available. Henry Ford Health System has a stable population especially within oncology since they are the largest healthcare insurer in the region and are a referral center, thus the drop-out rate is minimal. The study was approved by the Henry Ford Health System IRB.

Since physicians do not routinely assess loss of muscle in usual clinical practice, different definitions of cachexia were developed to capture other possible features of cachexia (weight loss) as well as diagnosis or treatment of conditions associated with cachexia (e.g., anorexia, anabolic steroids, and growth hormone) [13, 14]. The definitions were chosen to provide a range for how cachexia may be identified among cancer patients in clinical practice. Cachexia could occur at time of the cancer diagnosis or after. Also, patient identification for cachexia was done independently for each definition so some patients may be identified as having cachexia in more than one definition. For example, a patient may have a diagnosis of 799.4 and have ≥5% weight loss. 

Body weight was abstracted from the electronic medical records and percent change in weight was computed for patients with serial weight measures postcancer diagnosis. Due to the sporadic recording of weight, weight change was computed from the time of the first weight measure after cancer diagnosis up to death or end of study. This method provided a varying time period over which weight loss was observed and the focus of the analysis was on the presence of weight loss postdiagnosis rather than the severity or rapidness of onset. A threshold of 5% body weight loss was used since several studies have used this as a definition of cachexia [13, 16, 17]. Major comorbid conditions including stroke, coronary heart disease, peripheral vascular disease, heart failure, hypertension, chronic pulmonary disease, osteoarthritis, osteoporosis, and diabetes were abstracted from the patient's record to assess current health status. These conditions were selected since they were known to impact physical health, functioning, and activities of daily living which may result in loss of muscle mass or weight unrelated to their cancer. 

### 2.1. Statistical Analyses

Analyses were conducted for all cancer types combined. The proportion of patients for categorical variables and mean and standard deviation for continuous variables were computed for patients with cachexia compared with patients without cachexia (no ICD-9 diagnostic code, no prescription medications, and no weight loss ≥5%). Number of patients with cachexia (from 2000 to 2006) was estimated for each of the 4 definitions. The number of patients with cachexia was the numerator and the number of patients with the selected cancers was the denominator in computing proportions with cachexia. The length of follow up after cancer diagnosis in which to identify cachexia may differ between patients diagnosed in 1999 and those diagnosed in 2000 or later. Thus, the identification of cachexia did not start until 2000 to adjust for duration of cancer and follow-up time before cachexia was noted. Patients identified as having cachexia for each definition were compared with patients who did not meet any of the 4-definition criteria (no cachexia). Comparisons were made using *t*-tests for means and chi-square tests for proportions. No statistical adjustment was made for multiple comparisons. Percent agreement (proportion of concordant results among all tested) between different cachexia definitions was computed to assess the concordance between any 2 definitions. Since a gold standard definition was not available, percent agreement (instead of sensitivity, specificity, or predictive value) was used.

## 3. Results

There were 8541 cancer patients who were diagnosed with cancer between January 1999 and December 2004 and included in the study. Of the total sample, 39% of patients had prostate cancer, 25% breast, 15% lung, 11% colorectal, 3% head/neck, 3% pancreatic, 2% liver, 2% gastric, and 1.4% esophageal cancer. The rank order of cancer type within the Henry Ford tumor registry is the same as the SEER registry for 2000–2004 [16]. Mean age for eligible cancer patients was 63.6 (11.8) years, 60% were men, 55% were Caucasian, and 36% were African-American. Less than one-third of patients (29%) died during the study period.

### 3.1. Cachexia Based on ICD-9 Diagnostic Codes

There were 205 out of 8541 (2.4%) cancer patients with an ICD-9 code of 799.4 for cachexia during the follow-up period from cancer diagnosis to end of the study. The average age of patients with the diagnostic code for cachexia was 66 years and 34% were men (Table 1). Patients with the cachexia diagnostic code had significantly more comorbidity, especially coronary heart disease, heart failure, hypertension, and COPD than patients without cachexia (without any of the cachexia definition criteria) (*P* < .01) (Table 1). The largest proportion of patients with a cachexia diagnostic code had lung cancer (40.5%) and significantly more patients had lung, esophagus, gastric, or head/neck cancer compared with patients without cachexia. Cachexia was diagnosed in more patients with regional (49%) or distant (31%) metastatic cancer than in situ or local stages. Approximately 13% of patients with esophageal cancer had a diagnostic code for cachexia; less than 10% of the other cancer types had the diagnostic code.

The expanded ICD-9 diagnostic code definition including codes for cachexia, anorexia, abnormal weight loss and feeding difficulties identified 467 patients out of 8541 (5.5%) patients. Similar to the cachexia diagnostic code only, patients with the expanded code definition were mostly women (63%), mean age of 67 years, with substantial cardiovascular comorbidity (Table 1). A significantly greater proportion of cachexia patients had each of the comorbid conditions compared with patients without cachexia, *P* < .01 (Table 1). Significantly more patients with cachexia had esophageal, gastric, head/neck or lung cancer compared with patients without cachexia, *P* < .01. Significantly more patients with cachexia had regional (41%) or distant (21%) tumor stages than patients without cachexia (24% regional and 12% distant), *P* < .01. Approximately 21% of patients with esophageal cancer, 17% of patients with head/neck cancer, and 16% of patients with gastric cancer had cachexia based upon the expanded diagnostic codes definition. Less than 10% of patients with breast, colorectal, liver, lung, pancreatic, or prostate cancer had cachexia using this definition.

### 3.2. Cachexia Based upon Prescription Medication Use

Using the definition of prescription for megestrol acetate, oxandrolone, somatropin or dronabinol, 546 (6.4%) patients were identified as having cachexia. Significantly more patients taking a medication for possible treatment of cachexia were men and had cardiovascular disease, COPD, and diabetes compared with patients without cachexia, *P* < .01 (Table 1). For patients taking cachexia-related medications, 34% had lung, 21% had breast, and 16% had prostate cancer. A significantly greater proportion of the patients taking cachexia-related medications had regional or distant stage tumors compared with patients without cachexia, *P* < .01.

### 3.3. Cachexia Based upon ≥5% Weight Loss

Among the 8541 cancer patients, 65% of patients had body weight recorded in the electronic medical record and 3343 (39% of total) patients had 2 or more weights for the computation of weight loss after cancer diagnosis. Among the patients with 2 or more weight measurements, 1257 had ≥5% weight loss, which represents 14.7% of the total sample of 8541 and 37.6% of patients with ≥2 weight measurements. The mean time over which weight loss was observed was 655 (538) days, with a median of 487 days and interquartile range of 236–942. There were significantly more patients in the ≥5% weight loss/cachexia group with cardiovascular disease and diabetes compared with patients without cachexia by any definition, *P* < .01 (Table 1). The largest proportion of patients with ≥5% weight loss (*n* = 1257) had breast cancer (31%) or prostate cancer (29%). Patients with ≥5% weight loss were more likely to have regional (*P* < .01) but not distant stage tumors compared with patients without cachexia. Approximately 20% of patients with gastric (*n* = 171) or head/neck cancers (*n* = 256) had ≥5% weight loss, and 19% of the 2135 patients with breast cancer and 16-17% of patients with colorectal (*n* = 939), esophageal (*n* = 120), or liver (*n* = 171) cancer had ≥5% weight loss after their cancer was diagnosed and before the study ended.

The proportion of cancer patients with cachexia varied across cancer type and by cachexia definition (Table 2). As low as 0.8% of breast cancer patients were identified as having a diagnosis of cachexia (ICD-9 code of 799.4) but as many as 19% of breast cancer patients had significant weight loss (≥5%). Approximately 19-20% of patients with gastric cancer had cachexia based on the prescription medication and weight loss definitions but only 8% had received a diagnostic code for cachexia.

### 3.4. Cachexia by Any Definition

Cancer patients who met the criteria for any of the four cachexia definitions were identified and 1975 (23%) patients met at least one of the definitions for cachexia. Comparing patients with cachexia using any one of the definitions with cancer patients without cachexia, cachexia patients were older and greater percentages were men and had comorbid conditions compared with the patients without cachexia (Table 1). The largest proportion of cachexia patients had breast (27%), prostate (26%), or lung (20%) cancer. Over 41% of patients with esophageal or gastric cancer, 37% of patients with head/neck cancer, 35% of patients with pancreatic cancer and 31% of patients with lung cancer met at least one of the cachexia definitions.

**Figure 1:**
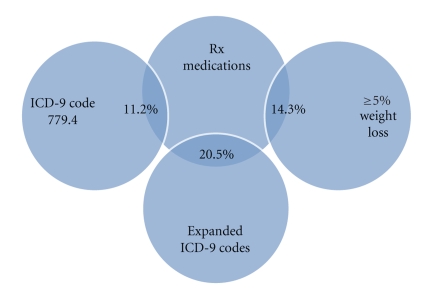
Pair-wise overlap between cachexia definitions and prescription medication definition.

**Figure 2:**
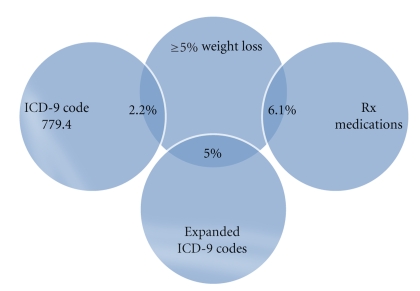
Pair-wise overlap between cachexia definitions and weight loss definition.

The amount of overlap between cachexia definitions was limited. Less than 22% of patients were classified as having cachexia based on prescription medications and either an ICD-9 diagnostic code or ≥5% weight loss (Figure 1). The overall percent agreement between the medication-defined cachexia and ICD-9 cachexia diagnostic code only definition was 93%, 91% for the extended diagnostic codes, and 81% for the weight loss definition. Less than 7% of patients were classified as having cachexia based on weight loss and either an ICD-9 code or prescription medication (Figure 2). The overall percent agreement between the weight loss definition and ICD-9 code for cachexia only was 84%, 81% for the extended diagnostic codes, and 81% for the medications definition. By definition, all patients with an ICD-9 code of 799.4 were included in the first two definitions (ICD-9 code of 799.4 and the expanded codes of cachexia, anorexia, or feeding difficulties).

## 4. Discussion

This study determined that the proportion of patients with cachexia varied considerably depending upon the definition employed, from 2.4% with the cachexia only diagnostic code to 15% with the weight loss definition. Additionally, up to 23% of cancer patients may have cachexia at some point after their cancer diagnosis as determined by any of the four definitions. The ≥5% weight loss definition identified the most patients with cachexia (15%) and weight loss is considered a hallmark symptom of cachexia [13] but not the only symptom. These findings highlight the need for a unified definition of cachexia since the proportion of patients identified varied widely and this variation would make it difficult to estimate the need for resources to identify and treat patients with cachexia. Work is underway to develop a unified definition of cachexia [13]. Also, the proportions identified with cachexia from each definition and the combined definition may underrepresent the true frequency of cachexia among cancer patients. The possible underestimation of cachexia may be due to several factors including limited therapeutic interventions for cachexia which may lead to it not being coded and the lack of weight loss data prior to the cancer diagnosis. In a study of 119 patients with advanced gastrointestinal cancer, investigators found that 81% had weight loss of >5% over 3 months [9]. Similarly, in 3000 cancer patients from the Eastern Cooperative Oncology Group clinical trials, weight loss of >5% was observed in 85% of gastric, 83% in pancreatic, 61% of nonsmall cell lung, 57% of small cell lung, and 54% of colon cancer patients [7]. The proportion of patients with cachexia in the present study was lower than those found in the Eastern Cooperative Oncology Group [7]. However, the Oncology Group estimated incidence rates of weight loss shortly after diagnosis and over 2 to 6 months before receiving treatment and the present study estimated proportion of patients with cachexia of all tumor stages and treatments. Also, the present study was among US patients versus United Kingdom patients and data capture of weight and weight loss may not be as consistent in the US as it is in a national healthcare system.

In comparing the proportions of patients with cachexia observed in the present study across the different definitions, it is apparent that a diagnosis of cachexia is given to a minority of patients (2.4%). Expanding the diagnostic codes to other conditions that may be surrogates for cachexia (i.e., anorexia, abnormal weight loss, and feeding difficulties) doubled the estimate for cachexia but did not approach the proportion based on ≥5% weight loss. This discrepancy may confirm anecdotal information that suggests that physicians may diagnose but not code for billing purposes abnormal weight loss among their cancer patients. Physicians do not receive any additional reimbursement for patients with abnormal weight loss so they may not include the weight loss in the diagnostic codes for billing and therefore it does not appear in the claims databases. Additionally, diagnostic codes may be used only in extreme cases (those appearing to be visually wasting) towards end of life as opposed to earlier diagnosis at a time when the course of the disease can be changed through interventions.

Cachexia also may not be widely treated since only 6.4% of patients were taking prescription hormones, anabolic steroids, or appetite stimulants and there is no FDA approved treatment for cachexia [13, 14]. Physicians may believe that available treatments are not effective or appropriate for long-term use and hence not used; therefore, this definition may not be a good surrogate for identifying cachexia. Data demonstrating the effectiveness of pharmacologic agents like androgens and growth hormone in treating cachexia is lacking [13].

Given the large variation in estimates across cachexia definitions, a standard, operational definition of cachexia is needed [13, 14]. Depending upon the definition of cachexia, the burden of cachexia in terms of mortality, health-related quality of life, healthcare resource utilization, and economics to health plans and society will vary widely making it difficult to identify and manage patients with cachexia. Without a standard definition, physicians are unlikely to identify, diagnose, and treat all cases of cachexia, which leaves patients vulnerable to higher mortality, and decreased quality of life [13, 14]. The lack of a standard definition also hinders healthcare decision makers' ability to plan appropriate resources and treatment for cachexia patients. For cancer patients, the diagnosis and treatment of cachexia will become important goals to improve morbidity, mortality and quality of life [13]. Once a unified definition of cachexia is developed, further research will be needed to determine the prevalence of cachexia among cancer patients.

When cachexia patients were compared with patients without cachexia, cachexia patients had more comorbid conditions, especially CHD, heart failure, hypertension, and COPD. This may indicate that cachexia patients have multiple health conditions including their cancer that may lead to a weakened state which may cause the cachexia to be diagnosed or exacerbate it. Also, patients with cachexia had more distant metastatic tumors compared with patients without cachexia.

The present study has limitations that should be considered. The study population included only individuals seeking care at the Henry Ford Health System and cancer patients seeking care at Henry Ford may not be representative of all cancer patients. Thus, caution in generalizing the findings beyond the Henry Ford Health System is needed. However, this is the largest study and the first US study of cachexia using available clinical data. Patients who had a shorter follow-up time for estimating cachexia (i.e., dropped out of Henry Ford Health System) may be different from those with complete follow-up time, but Henry Ford Health System has a stable patient population and is one of the largest referral centers in the region. Data prior to the cancer diagnosis was not available so some prevalent cases of cachexia that developed prior to the cancer diagnosis may not have been included. This may have affected the weight loss definition of cachexia more than the other definitions because weight loss prior to the cancer diagnosis could not be assessed. For patients with more than one weight measurement, weight loss was computed from the first weight measure after cancer diagnosis to the second weight measure. Thus, the time period for assessing weight loss varied over time for patients with weight loss ≥5%. The clinical course of a patient losing ≥5% of weight over 1–2 months is very different than a patient losing the weight over 12 or more months; however, this study focused on the presence of weight loss and not the time course or severity of the weight loss. Also, weight loss due to reversible causes like treatment side effects or mechanical GI obstruction could not be ruled out and was classified as cachexia in this study if it was ≥5%. Since there is no widely used clinical definition of cachexia and physicians do not typically measure muscle loss in cancer patients, cachexia is likely to be an underutilized diagnosis and under-coded ICD-9 code. Thus, the study definitions of cachexia by ICD-9 codes and prescription medications included anorexia, which may not be true clinical cases of cachexia; thus our estimates of cachexia may be an over-estimation. Also, there may be a survival bias across the definitions of cachexia so the identification of cachexia may be confounded by the length of follow up. For example, patients in the weight loss definition were followed at least until 2-weight measures were recorded compared with patients with a diagnostic code for cachexia.

In conclusion, each cachexia definition provided a different estimate of the proportion of patients with cachexia. If all four definitions are utilized to identify cachexia patients, approximately 23% of cancer patients will have cachexia at some time after their cancer diagnosis. This does not include the proportion of patients that had unintentional weight loss leading up to the cancer diagnosis. A standard, well-accepted definition of cachexia is needed to improve clinical research and diagnosis and treatment of cachexia, and work is underway [13]. Unintentional weight loss of ≥5% may be the best criteria currently to alert clinicians that detailed follow up for cachexia may be needed after ruling out reversible causes of weight loss.

## Figures and Tables

**Table 1:** Characteristics of cancer patients identified as having cachexia based on the different definitions and compared with cancer patients without cachexia.

Characteristics	Cancer patients with cachexia ICD-9 code (799.4) *n* = 205	Cancer patients with cachexia ICD-9 codes (cachexia, anorexia, abnormal weight loss, or feeding difficulties) *n* = 467	Cancer patients with prescription for megestrol acetate, oxandrolone, somatropin or dronabinol *n* = 546	Cancer patients with weight loss ≥5% *n* = 1257	Cancer patients with cachexia by any definition *n* = 1975	Cancer patients without cachexia^† ^ *n* = 6,566
Age, years, mean	65.6*	66.9**	64.4*	66.3**	65.8**	63.0
Gender, men, %	34.1	37.0	46.7**	49.7**	46.9**	38.4

Comorbidities, %						

Stroke	3.9	11.9**	7.9**	4.9	6.5**	3.8
CHD	27.3**	35.1**	33.7**	26.8**	29.5**	16.7
Peripheral vascular disease	4.4	7.9**	4.8**	5.1**	5.3**	2.5
CHF	24.9**	28.5**	21.6**	17.6**	20.5**	10.0
Hypertension	54.2**	67.0**	60.8**	56.1**	58.7**	43.6
COPD	38.1**	32.6**	26.2**	18.1**	21.6**	11.4
Osteoarthritis	11.7	25.1**	15.9**	19.1**	18.9**	11.7
Osteoporosis	3.9	5.8**	5.1**	5.0**	4.8**	3.0
Diabetes	16.6	24.4**	24.0**	26.3**	25.9**	17.2

Cancer type, %						

Breast	8.8 **	13.9**	20.5	31.3**	26.5*	24.2
Colorectal	11.2	11.8	10.3	11.8	11.7	10.3
Esophagus	7.3**	5.1**	2.9**	1.5	2.5**	1.0
Gastric	5.9**	4.7**	5.0**	2.2*	3.0**	1.3
Head/neck	7.3**	9.0**	2.8	3.9**	4.6**	2.4
Liver	2.4	2.1	1.1	2.1	1.9	1.8
Lung	40.5**	26.8**	33.5**	15.6	20.3**	13.6
Pancreas	3.9	3.4	7.9**	2.2	3.9**	2.2
Prostate	12.7**	23.1**	16.1**	29.4**	25.7**	43.3

Tumor stage, %						

In situ	0.5**	2.4**	3.5**	8.4	6.6	6.9
Local	16.6**	33.0**	25.5**	50.4*	42.3**	54.3
Regional	48.8**	41.1**	39.2**	28.2**	31.9**	24.3
Distant	31.2**	21.1**	28.9**	10.7	16.9**	12.1
Unknown	2.9	2.4	2.9	2.3	2.4	2.4

^†^No cachexia based on any of the 4 definitions (no ICD-9 diagnostic codes, prescription medications or ≥5% weight loss). *P < .05, **P < .01 chi-square test or *t*-test comparing each cachexia definition to cancer patients without cachexia. Stroke/TIA ICD-9 code = 435.xx, 436.xx, CHD = 410.xx, 411.xx, 412.xx, 414.xx, 429.2, 440.xx or procedures = 36.0x, 36.1x, 38.12–38.14, 38.16, 38.18, 38.34, 38.44, 39.22–39.26, 39.50 or CPT codes = 33510–33516, 33517–33545, 33572, 33877, 34800–34832, 35061–35103, 35301, 35311–35381, 35390, 35450–35481, 35482–35495, 35500–35683, 92980–92981, 92982–92984, 92995–92966, PVD = 443.xx, 39.29, CHF = 428.xx, hypertension = 401.xx, 402.xx, 404.xx, osteoarthritis = 715.xx, osteoporosis = 733.00–733.09, 737.40, 737.41, COPD = 493.20–493.22, 496, 491.00–491.21, 491.8, 491.9, diabetes = 250.xx, 362.0x, 366.41.

**Table 2:** Proportion of cancer patients with cachexia by cancer type.

Cancer type	Cancer patients with cachexia ICD-9 code only	Cancer patients with any cachexia ICD-9 code	Cancer patients taking prescription medication indicative of cachexia	Cancer patients with ≥5% weight loss	Cancer patients with any one of the cachexia definitions
Breast, *n* = 2112	0.8%	3.1%	5.3%	18.6%	24.8%
Colorectal, *n* = 905	2.5%	6.1%	6.2%	16.4%	25.5%
Esophagus, *n* = 117	12.8%	20.5%	13.7%	16.2%	41.9%
Gastric, *n* = 142	8.4%	15.5%	19.0%	19.7%	41.5%
Head/neck, *n* = 246	6.1%	17.1%	6.1%	19.9%	37.0%
Liver, *n* = 153	3.3%	6.5%	3.9%	17.0%	24.2%
Lung, *n* = 1291	6.4%	9.7%	14.2%	15.2%	31.1%
Pancreas, *n* = 221	3.6%	7.2%	19.5%	12.7%	34.8%
Prostate, *n* = 3354	0.8%	3.2%	2.6%	11.0%	15.1%

## References

[1] Tisdale MJ (2002). Cachexia in cancer patients. *Nature Reviews Cancer*.

[2] Strasser F, Bruera ED (2002). Update on anorexia and cachexia. *Hematology/Oncology Clinics of North America*.

[3] Argiles JM, Busquets S, Felipe A, Lopez-Soriano FJ (2006). Muscle wasting in cancer and ageing: cachexia versus sarcopenia. *Advances in Gerontology*.

[4] Giordano KF, Jatoi A (2005). The cancer anorexia/weight loss syndrome: therapeutic challenges. *Current Oncology Reports*.

[5] National Cancer Institute http://www.cancer.gov/cancertopics/pdq/supportivecare/nutrition/healthprofessional.

[6] O'Gorman P, McMillan DC, McArdle CS (1999). Longitudinal study of weight, appetite, performance status, and inflammation in advanced gastrointestinal
cancer. *Nutrition and Cancer*.

[7] Dewys WD, Begg C, Lavin PT (1980). Prognostic effect of weight loss prior to chemotherapy in cancer patients. Eastern Cooperative Oncology Group. *American Journal of Medicine*.

[8] Bruera E (1997). ABC of palliative care: anorexia, cachexia, and nutrition. *British Medical Journal*.

[9] O'Gorman P, McMillan DC, McArdle CS (1998). Impact of weight loss, appetite, and the inflammatory response on quality of life in gastrointestinal cancer
patients. *Nutrition and Cancer*.

[10] Persson C, Glimelius B (2002). The relevance of weight loss for survival and quality of life in patients with advanced gastrointestinal cancer treated with palliative chemotherapy. *Anticancer Research*.

[11] Scott HR, McMillan DC, Brown DJF, Forrest LM, McArdle CS, Milroy R (2003). A prospective study of the impact of weight loss and the systemic inflammatory response on quality of life in patients with inoperable non-small cell lung cancer. *Lung Cancer*.

[12] Andreyev HJ, Norman AR, Oates J, Cunningham D (1998). Why do patients with weight loss have a worse outcome when undergoing chemotherapy for gastrointestinal malignancies?. *European Journal of Cancer*.

[13] Evans WJ, Morley JE, Argilés J (2008). Cachexia: a new definition. *Clinical Nutrition*.

[14] Lainscak M, Filippatos GS, Gheorghiade M, Fonarow GC, Anker SD (2008). Cachexia: common, deadly, with an urgent need for precise definition and new therapies. *The American Journal of Cardiology*.

[15] Springer J, von Haehling S, Anker SD (2006). The need for a standardized definition for cachexia in chronic illness. *Nature Clinical Practice Endocrinology and Metabolism*.

[16] Palesty JA, Dudrick SJ (2003). What we have learned about cachexia in gastrointestinal cancer. *Digestive Diseases*.

[17] Maltoni M, Nanni O, Scarpi E, Rossi D, Serra P, Amadori D (2001). High-dose progestins for the treatment of cancer anorexia-cachexia syndrome: a systematic review of randomised clinical trials. *Annals of Oncology*.

